# Supplementary material for: Conserved and tissue-specific immune responses to biologic scaffold implantation
Source: bioRxiv. 2023 Aug 17:2023.08.15.553390. Preprint. [Version 1] doi: 10.1101/2023.08.15.553390 (PMC10560402; doi:10.1101/2023.08.15.553390)
Supplement: Supplement 1 [file media-1.pdf]

SUPPLEMENTARY FIGURES AND LEGENDS

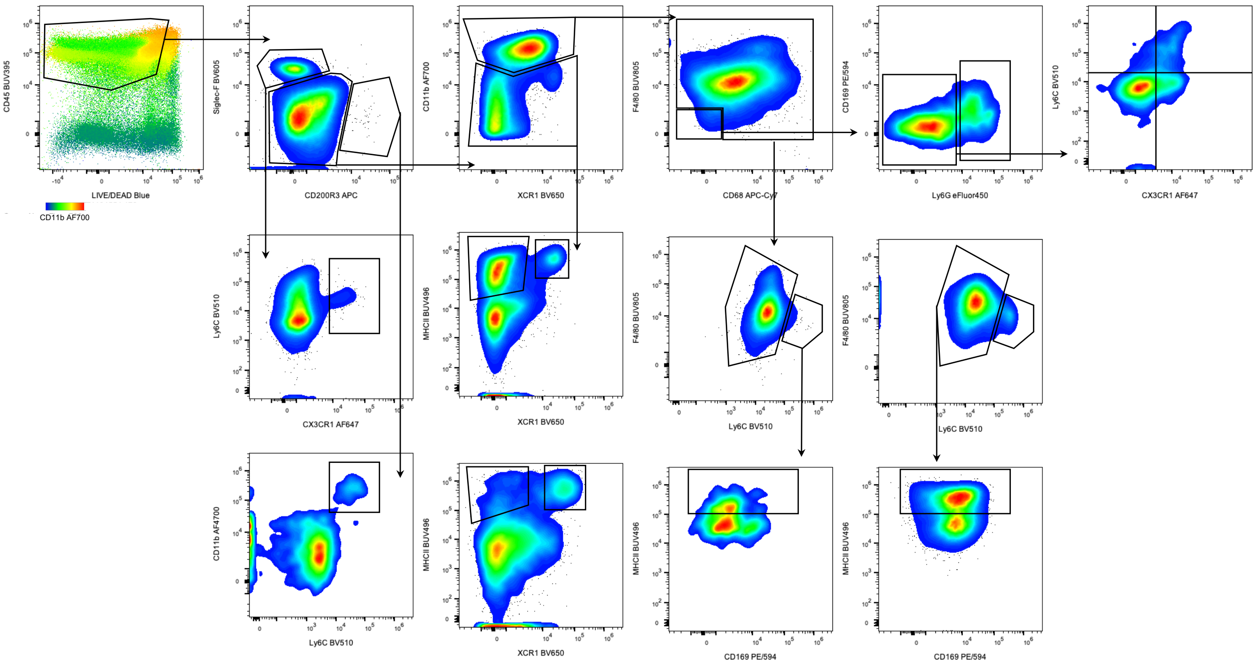

SUPPLEMENTARY FIGURE 1 | Flow cytometry gating strategy for myeloid phenotyping. Example gating is shown from intraperitoneal and volumetric muscle loss implant samples. FSC-A x SSC-A and FSC-A x FSC-H were used to discriminate debris and doublets before gating on live immune cells (FSC = forward scatter, SSC = side scatter, A = area, H = height). Modified from a preprinted manuscript [19].

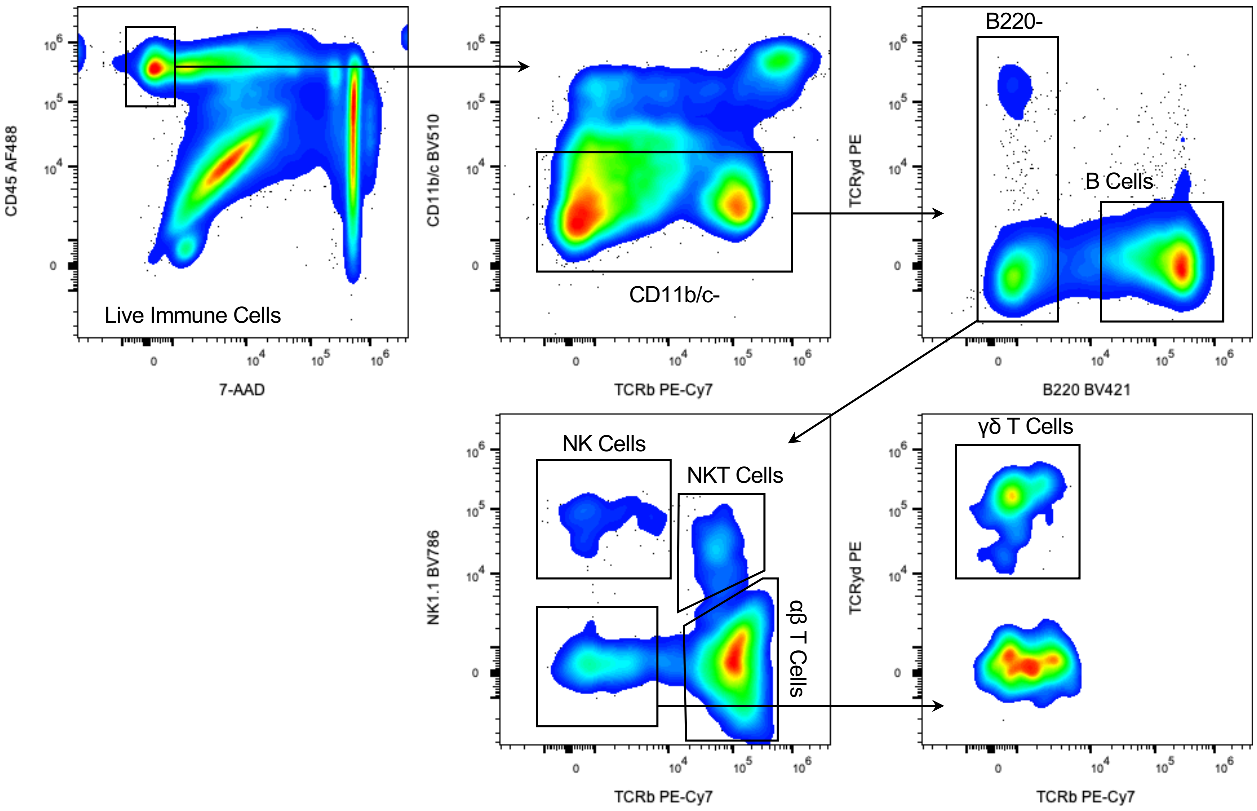

SUPPLEMENTARY FIGURE 2 | Flow cytometry gating strategy for lymphoid phenotyping. Example gating shown from intraperitoneal implant sample. FSC-A x SSC-A and FSC-A x FSC-H were used to discriminate debris and doublets before gating on live immune cells (FSC = forward scatter, SSC = side scatter, A = area, H = height).

Marker	Target	Clone	Fluorophore	Vendor	Dilution Factor	Bead or Cell Control
CD45	Leukocytes	30-F11	BUV395	BD	1:100	PBMC
Viability	Dead Cells		LIVE DEAD Blue	Thermo	1:1000	RAW264.7
MHCII	APC	2G9	BUV496	BD	1:400	PBMC
CD1d	Lipid Ag Present	1B1	BUV563	BD	1:200	Beads
F4/80	Macrophage	T45-2342	BUV805	BD	1:100	Beads
CD206	M2 Mac	C068C2	BV421	Biolegend	1:100	Beads
Ly6G	Neutrophil	1A8	eFluor 450	Thermo	1:200	Beads
CD103	DC	M290	BV480	BD	1:200	Beads
Ly6C	Monocyte	HK1.4	BV510	Biolegend	1:200	PBMC
CD11c	DC	N418	BV570	Biolegend	1:200	Beads
Siglec-F	Eosinophil	E50-2440	BV605	BD	1:200	Beads
XCR1	DC	ZET	BV650	Biolegend	1:400	Beads
CD11b	Myeloid	M1/70	Alexa Fluor 700	Biolegend	1:400	PBMC
CD8α	DC	53-6.7	Spark Blue 550	Biolegend	1:100	PBMC
CD301b	Macrophage	URA-1	PE/Cy7	Biolegend	1:400	PBMC
CD86	M1 Mac	A17199A	PE	Biolegend	1:400	PBMC
CD169	Tissue Resident	3D6.112	PE/Dazzle594	Biolegend	1:400	Beads
CCR7	M1 Mac	4B12	PE-Cy5	Biolegend	1:200	PBMC
CD200R3	Baso/Mast Cell	Ba13	APC	Biolegend	1:200	Beads
CX3CR1	Pre-M2	SA011F11	Alexa Fluor 647	Biolegend	1:400	PBMC
CD68	Macrophage	FA-11	APC-Cy7	Biolegend	1:100	Beads

SUPPLEMENTARY TABLE 1 | Flow cytometry antibodies for the myeloid phenotyping panel. Modified from a preprinted manuscript [19].

Marker	Target	Clone	Fluorophore	Vendor	Dilution Factor	Bead or Cell Control
CD45	Immune	I3/2.3	AF488	BD	1:100	Beads
Viability	Dead Cells		7-AAD	BD	Neat	PBMCs
TCR γδ	γδ T Cells	GL-3	PE	BD	1:100	Beads
TCRβ	αβ T cells	H57-597	PE/Cy7	BD	1:100	Beads
B220	B Cells	RA3-6B2	BV421	BD	1:100	Beads
CD11b	Myeloid	M1/70	BV510	BD	1:100	Beads
CD11c	Myeloid	HL3	BV510	BD	1:100	Beads
NK1.1	NK Cells	PK136	BV786	BD	1:100	Beads

SUPPLEMENTARY TABLE 2 | Flow cytometry antibodies for a lymphoid phenotyping panel.
Modified from a preprinted manuscript [19]
